# The role of neoadjuvant her2-targeted therapies in her2-overexpressing breast cancers

**DOI:** 10.3747/co.v16i5.510

**Published:** 2009-09

**Authors:** J. Lemieux, M. Clemons, L. Provencher, S. Dent, J. Latreille, J. Mackey, K.I. Pritchard, D. Rayson, Sh. Verma, Su. Verma, B. Wang, S. Chia

**Affiliations:** * Unité de recherche en santé des populations, Centre de Recherche du Centre Hospitalier affilié universitaire de Québec, Quebec City, QC; † Hôpital du Saint-Sacrement, Quebec City, QC; ‡ Princess Margaret Hospital, Toronto, ON; § Ottawa Hospital Cancer Centre, Ottawa, ON; || Centre Intégré de cancérologie de la Montérégie and Réseau Cancer Montérégie, Longueuil, QC; # Cross Cancer Institute, Edmonton, AB; ** Sunnybrook Odette Cancer Centre, Toronto, ON; †† Atlantic Clinical Cancer Research Unit and QEII Health Sciences Centre, Halifax, NS; ‡‡ Remedy Communications Limited, Toronto, ON; §§ British Columbia Cancer Agency–Vancouver Centre, Vancouver, BC

**Keywords:** Neoadjuvant, breast cancer, her2-targeted therapy, trastuzumab

## Abstract

Women receiving neoadjuvant systemic therapy for primary operable or inoperable breast cancer can potentially benefit in a number of ways, but the main advantage, which has been consistently demonstrated, is improved tumour resectability. Given the improvement in outcomes with the adjuvant use of trastuzumab in patients with early-stage breast cancer positive for the human epidermal growth factor receptor 2 (her2), questions have been raised about the use of trastuzumab in the neoadjuvant setting. The present paper reviews the currently available data and outlines suggestions from a panel of Canadian oncologists about the use of trastuzumab and other her2-targeted agents in the neoadjuvant setting.

The panel focussed on

- the use of trastuzumab and other her2-targeted agents as neoadjuvant therapy in primary operable, locally advanced, and inflammatory breast cancer; and
- possible choices of chemotherapeutic regimens with trastuzumab.

The suggestions described here will continue to evolve as data from current and future trials with trastuzumab and other her2-targeted agents emerge.

## 1. INTRODUCTION

Chemotherapy produces similar survival gains whether given in the neoadjuvant or the adjuvant setting. A meta-analysis of randomized trials comparing neoadjuvant with adjuvant chemotherapy found no difference in distant disease recurrence [risk ratio (rr): 0.94; 95% confidence interval (ci): 0.83 to 1.06] or overall survival rate (rr: 1.00; 95% ci: 0.90 to 1.12) 1. Notably, however, most of the patients in the relevant trials had operable cancer at randomization.

In the clinical setting, neoadjuvant therapy has several potential advantages over standard adjuvant therapy. First, for operable tumours, the use of neoadjuvant therapy increases the rate of breast-conserving surgery 2,3, with the caveat that neoadjuvant therapy is associated with an increase in locoregional recurrence (rr: 1.22; 95% ci: 1.04 to 1.43), especially in younger women (in whom tamoxifen was not given at the time that the trial was conducted) and in women for whom a mastectomy was initially proposed but was changed to breast-conserving surgery after neoadjuvant chemotherapy 2. Second, given that a pathologic complete response (pcr) is a surrogate marker for improved clinical outcome 2, neoadjuvant therapy provides an opportunity to undertake correlative research by allowing *in vivo* assessment of tumour response to particular drug regimens 3. Lastly, from a practical viewpoint, neoadjuvant therapy allows patients to start treatment earlier if surgical wait times are too long. Neoadjuvant therapy also has its disadvantages, however. First, there is about a 5% chance that tumour progression may occur during neoadjuvant treatment, thereby delaying definitive local therapy 4. In addition, neoadjuvant treatment prevents definitive pathologic staging.

Locally advanced breast cancer (labc) and inflammatory breast cancer (ibc) are, by definition, inoperable, and neoadjuvant therapy is therefore the sole treatment option. Chia *et al.* 5 and Anderson *et al.* 6 both described labc as stage iii disease (excluding T4d tumours), with a heterogeneous clinical phenotype that ranges from neglected low-grade estrogen receptor (er)–positive to rapidly progressing er-negative breast cancer. On the other hand, ibc (T4d) appears to have a different epidemiology, is typically diagnosed at a younger age, and is generally associated with higher tumour grade, negative er status, and increased angiogenesis and lymphangiogenesis; historically, ibc has a worse survival rate 5–8.

The human epidermal growth factor receptor 2 (her2) is a transmembrane tyrosine kinase receptor protein that is part of the her family of growth factor receptors (her1–her4). The her2 receptor is involved in cell–cell and cell–stroma communication primarily through signal transduction involving the Ras/mitogen-activated protein kinase pathway and the phosphoinositide 3 kinase (pi3k)/Akt pathway 9. These signals ultimately promote cell proliferation, survival, and motility. Amplification of the her2/*neu* gene and resulting overexpression of the her2 protein occurs in approximately 15%–20% of invasive primary breast cancers 10,11. Compared with her2-negative cancers, early-stage breast cancer with a her2 alteration is associated with more aggressive disease and a higher risk of relapse 12–16.

Trastuzumab is a monoclonal antibody that binds to the extracellular domain of her2. Several mechanisms of action underlie the antitumour effects of trastuzumab. Trastuzumab blocks her2- activated cell signalling, thereby reducing cell proliferation and restoring a capacity for apoptosis by inhibiting the pi3k/Akt pathway 17–19, which increases cellular sensitivity to chemotherapy and radiotherapy 20. Trastuzumab has been shown to inhibit her2-regulated angiogenesis 17,21–23 and, in preclinical models, to recruit the immune system through antibody-dependent cellular cytotoxicity, triggering activation of natural killer cell–mediated apoptosis 24–26. Trastuzumab has also been shown to prevent the formation of p95^her^^2^ (a truncated, active form of her2), which may lead to inhibition of tumour development 17,27.

Trastuzumab is now the standard of care in the adjuvant setting. A meta-analysis 28 of the five randomized controlled trials in the adjuvant setting [North Central Cancer Treatment Group (ncctg) 9831, National Surgical Adjuvant Breast and Bowel Project (nsabp) B31, the Herceptin^a^ Adjuvant (hera) trial, the Finland Herceptin (FinHer) trial, and Breast Cancer International Research Group 006] 29–32 comparing chemotherapy plus trastuzumab with chemotherapy alone in her2-positive disease observed a relative reduction in relapse of 47% (absolute reduction: 7.1%) and a relative reduction in mortality rate of 48% (absolute reduction: 2.5%) after a follow-up of 2–3 years.

The body of studies evaluating the use of her2- targeted agents in neoadjuvant therapy for both primary operable and primary inoperable her2- positive breast cancer is growing, and the present paper reviews the current evidence and outlines suggestions from a panel of Canadian oncologists about the neoadjuvant use of trastuzumab and other her2-targeted agents.

## 2. DEVELOPMENT OF PANEL SUGGESTIONS

The authors of this paper met in Toronto for a 1-day conference in June 2008. The panel reviewed the results of the latest trials using trastuzumab and other her2-targeted agents in the neoadjuvant, adjuvant, and metastatic disease settings. Based on trial information, suggestions for each setting were formulated. For neoadjuvant therapy, a draft manuscript reviewing the currently available data and outlining the suggestions from the panel was initially written by a medical writer (BW) and reviewed and revised by four panel members (JLe, MC, LP, SC). The final manuscript was reviewed and approved by the rest of the panel members (SD, JLa, JM, KIP, DR, ShV, SuV). Recent clinical trial results (available as of June 2009) were incorporated into the present document, and the conclusions discussed were updated as additional evidence became available.

Development of the Canadian advisory panel and the present manuscript were funded by an unrestricted educational grant from Hoffmann–La Roche Canada. The authors received an honorarium for attending the conference, but not for writing the manuscript. The authors are responsible for the content of the manuscript, with no restrictions set by the sponsor.

The panel focussed on

- the use of trastuzumab and other her2-targeted agents as neoadjuvant therapy in primary operable, locally advanced, and inflammatory breast cancer; and
- possible choices of chemotherapeutic regimens with trastuzumab.

The related clinical evidence is presented and discussed below. Key panel suggestions are followed by a discussion of the supporting evidence, the panel’s rationale for the suggestions, and any points on which consensus was lacking.

## 3. NEOADJUVANT TRIALS USING TRASTUZUMAB

Two recently reported randomized controlled prospective trials comparing chemotherapy with and without her2-targeted therapy in the neoadjuvant setting (Table I) are reviewed here.

### 3.1 MD Anderson Trial

In a small, two-arm, phase iii randomized prospective trial, Buzdar *et al.* compared 225 mg/m^2^ paclitaxel in a 24-hour continuous intravenous infusion every 3 weeks for 4 cycles, followed by fec [500 mg/m^2^ 5-fluorouracil (5fu) on days 1 and 4, 75 mg/m^2^ epirubicin on day 1, 500 mg/m^2^ cyclophosphamide on day 1] every 3 weeks for 4 cycles as neoadjuvant therapy without (p→fec) and with [h+(p→fec)] concurrent trastuzumab (4 mg/kg loading dose on day 1 of the first treatment cycle before paclitaxel, and 2 mg/kg maintenance dose weekly for 24 doses) 3. The study accrued 42 patients with histologically confirmed invasive, but noninflammatory, stage ii–iiia carcinoma of the breast. The primary objective was to demonstrate a doubled improvement in pcr—defined as no evidence of residual invasive cancer in both breast and axilla—with the addition of trastuzumab to chemotherapy. The trastuzumab-based treatment arm displayed a pcr improvement by a factor of 2.5 relative to chemotherapy alone (65.2% vs. 26.3%, *p* = 0.016). As a result of the superior response attributed to trastuzumab, the data analysis was presented to the MD Anderson Data Monitoring Committee. Based on recommendations by that committee, the protocol of the original study was changed to discontinue the chemotherapy-alone arm. Recruitment was continued in the h+(p→fec) arm only. A total of 45 patients received the h+(p→fec) regimen. The pcr rate for the updated combined trastuzumab treatment arm (*n* = 45) was also superior to chemotherapy alone (60% vs. 26.3%; 95% ci: 44.3% to 74.3%; Table I) 33. Notably, trastuzumab was given concomitantly with epirubicin. The median left ventricular ejection fraction (lvef) was 65% at baseline in the both trial arms; after 6 months, the median lvef was 65% (range: 55%–70%) in the (p→fec) arm, but 60% (range: 52%–70%) in the h+(p→fec) arm. No clinical cardiac dysfunction was reported; however, the sample size was small, and the follow-up duration was short.

### 3.2 Neoadjuvant Herceptin Trial

The international multicentre phase iii randomized noah (Neoadjuvant Herceptin) trial compared the efficacy of doxorubicin (60 mg/m^2^) and paclitaxel (150 mg/m^2^) every 3 weeks for 3 cycles, followed by paclitaxel alone (175 mg/m^2^) every 3 weeks for 4 cycles, followed by cyclophosphamide (600 mg/m^2^), methotrexate (40 mg/m^2^), and 5fu (600 mg/m^2^) every 4 weeks on days 1 and 8 for 4 cycles without (ap→p→cmf) and with [h+(ap→p→cmf)] concurrent trastuzumab [8 mg/kg loading dose, then 6 mg/kg every 3 weeks (concurrent with cycle 1 onward) continued to 52 weeks] as first-line therapy in patients with her2-overexpressing labc (including ibc) 34. A her2-negative third arm was also accrued as reference for the her2-overexpressing arms, but these patients were not given trastuzumab. Data presented here refer to the group of her2-positive patients (trastuzumab arm vs. no trastuzumab arm).

The primary endpoint was event-free survival (efs), which was defined as progression on therapy, relapse after surgery, or death from any cause. The secondary endpoints were pcr rate, overall survival, and safety and tolerability. In both her2-positive arms (with and without trastuzumab), 42%–43% of the patients presented with T4 noninflammatory breast cancer, 27% presented with ibc, and 30%–31% presented with N2 disease. In the intention-to-treat population (*n* = 227), patients receiving trastuzumab (*n* = 115) experienced a significantly improved 3-year efs rate relative to chemotherapy alone [70.1% vs. 53.3%; hazard ratio (hr): 0.56; 95% ci: 0.36 to 0.85; *p* = 0.006; Table I].

In addition, the pcr rate was significantly higher in the trastuzumab-containing treatment arm (43% vs. 23%, *p* = 0.002), and a trend toward improved overall survival was observed (hr: 0.65; 95% ci: 0.34 to 1.23; *p* = 0.18) 34. The rate of breast-conserving surgery nearly doubled in the trastuzumab arm (23% vs. 12.5%, *p* = 0.07) 36. In the subgroup of patients with her2-positive ibc (*n* = 61), the addition of trastuzumab to chemotherapy also significantly improved pcr (55% vs. 19%, *p* = 0.004) and total pcr (48% vs. 13%, *p* = 0.002) 35.

Neoadjuvant trastuzumab was well tolerated with reasonable cardiac safety. Only a 2.1% cumulative incidence of New York Heart Association (nyha) grade 3 congestive heart failure (chf) was observed in the trastuzumab arm 34 even though trastuzumab was delivered concurrently with doxorubicin (60 mg/m^2^) and paclitaxel (150 mg/m^2^). In contrast, the non-trastuzumab-containing group in her2-positive patients and the her2-negative group both reported no incidence of nyha grade 3 chf.

Although this study is very interesting, to date it has been published only in abstract form. No other randomized trials have compared trastuzumab with no trastuzumab treatment. However, a number of other ongoing trials have included trastuzumab as part of the treatment arm in the her2-overexpressing cohort.

The two trials presented next included concomitant (GeparQuattro) or sequential (techno) anthracyclines and trastuzumab.

### 3.3 GeparQuattro Trial

A multicentre prospective randomized three-arm trial studied the possible benefits of adding capecitabine, either concomitantly or sequentially, to docetaxel after 4 cycles of epirubicin (90 mg/m^2^) and cyclophosphamide (600 mg/m^2^) 37 (ec). Patients with her2-positive breast cancer received trastuzumab for 1 year, starting with ec in all three arms, thereby collecting data on the cardiac safety of trastuzumab and anthracyclines given concomitantly. Results presented in an abstract form showed only that neoadjuvant trastuzumab was well tolerated, with no cases of grade 4 congestive heart failure. No patients showed a decrease in lvef to below 45%. The pcr rate in a combined analysis of all three her2-positive treatment arms treated with concurrent trastuzumab was 45%. Investigators concluded that the addition of trastuzumab to chemotherapy was feasible without clinically relevant cardiotoxicity.

### 3.4 Taxol Epirubicin Cyclophosphamide Herceptin Neoadjuvant Trial

The multicentre phase ii single-arm Taxol Epirubicin Cyclophosphamide Herceptin Neoadjuvant (techno) trial (NCT00795899) examined the safety and efficacy of epirubicin (90 mg/m^2^) and cyclophosphamide (600 mg/m^2^) every 3 weeks for 4 cycles, followed by trastuzumab (8 mg/kg loading dose, followed by 6 mg/kg) and paclitaxel (175 mg/m^2^) every 3 weeks for 4 cycles as neoadjuvant therapy in patients with her2-positive primary breast cancer >2 cm or inflammatory disease 38. After surgery, patients continued trastuzumab (6 mg/kg) every 3 weeks for 12 cycles. The primary clinical endpoints were histopathologic response rates (breast and axilla) and overall cardiac toxicity; secondary endpoints were disease-free survival, overall survival, rate of breast conservation, and quality of life. This trial has been completed. In an abstract presented in 2006, the pcr rate was 20% 39.

## 4. NEOADJUVANT TRIALS USING LAPATINIB

Recently, Migliaccio *et al.* 19 performed two sequential single-arm neoadjuvant clinical trials in her2- overexpresing labc patients to compare the clinical efficacy and mechanism of action of trastuzumab with those of lapatinib, an oral epidermal growth factor receptor/her2 tyrosine kinase inhibitor. Patients in the single-arm trastuzumab trial were given a standard trastuzumab dose for the first 3 weeks followed by trastuzumab and standard docetaxel every 3 weeks for 12 weeks (*n* = 35). Patients in the lapatinib single-arm trial were given 1500 mg oral lapatinib daily for 6 weeks followed by trastuzumab and standard docetaxel every 3 weeks for 12 weeks (*n* = 49). Clinical efficacy of the trial that gave initial trastuzumab, as determined by the pcr rate [defined here as the complete disappearance of all invasive cancer or minute foci of residual disease (<0.5 cm) in breast], was 34% (11/32) as compared with 68% (23/34) in the trial that gave initial lapatinib. It must be noted that, because both trials used trastuzumab with docetaxel, the lapatinib trial was not a lapatinib-only design. Further study results showed that tumours with low pten predicted resistance to trastuzumab.

## 5. FUTURE STUDIES

Using www.clinicaltrials.gov, we found 14 randomized clinical trials for her2-positive breast cancer in the neoadjuvant setting (Table II). Most of these trials used pcr as the primary endpoint.

Eight trials compared trastuzumab with lapatinib or a combination of both agents. One trial (NeoSphere) is currently examining the safety and efficacy of the various combinations of trastuzumab and pertuzumab, another monoclonal antibody that targets her2, in the presence or absence of chemotherapy for labc or ibc. As a complement to trastuzumab, pertuzumab inhibits the her2-mediated signalling pathway by binding to a different epitope on the her2 extracellular domain, thereby preventing her2 dimerization with other members of the her2 family 40. *In vitro* evidence suggests that these two agents act synergistically to inhibit the survival of breast cancer cells 41, suggesting that the combination of these two agents would be a more effective therapeutic strategy.

The American College of Surgeons Oncology Group (acosog) Z1041 trial is examining the sequence of fec_75_–paclitaxel plus trastuzumab, in which fec_75_ is given either before or after the paclitaxel and trastuzumab. Other trials are testing new agents such as everolimus.

The German Breast Group 44 trial (GeparQuinto) will randomize 2547 patients to six arms: one without neoadjuvant targeted therapy, one with bevacizumab, one with only trastuzumab and no chemotherapy, one with everolimus, one with chemotherapy plus trastuzumab, and one with chemotherapy plus lapatinib. These large clinical trials may answer many clinical questions, such as “Is it better to give a combination of targeted agents rather than one agent?”

## 6. PANEL SUGGESTIONS FOR TREATMENT IN NEOADJUVANT THERAPY

### 6.1 HER2-Targeted Agents as Neoadjuvant Therapy in HER2-Positive Primary Operable LABC or IBC

#### Suggestion

Currently, the panel felt that trastuzumab is standard in the neoadjuvant setting in combination with chemotherapy in patients with her2-overexpressing breast cancer when a clinical decision has been made to treat with neoadjuvant chemotherapy. Other her2-targeting agents such as lapatinib and pertuzumab should be given only as part of a clinical trial.

#### Discussion

The addition of trastuzumab in neoadjuvant systemic therapy clearly increases pcr in primary operable breast cancer, and a growing body of clinical evidence shows a similar result in labc and ibc^3^,^33^–^35^,^37^. Furthermore, increased pcr after preoperative therapy has been shown to be a surrogate marker for long-term disease-free survival and overall survival in some trials 2,3. However, in the nsabp B27 trial, despite increased pcr, no survival advantage was shown with the addition of docetaxel to an anthracycline-based regimen. Investigation of new agents in combination with conventional systemic therapies in the preoperative or neoadjuvant setting can provide tissue sampling to assess markers of *in vivo* tumour responses. As well, pcr can be used as a surrogate, allowing for testing of new interventions in smaller phase ii trials before they are studied in larger phase iii clinical trials, which require a larger sample size and longer follow-up.

The latest and strongest evidence relies on the final study results of the noah trial. Here, the addition of trastuzumab to chemotherapy in her2-positive patients significantly improved event-free survival at 3 years relative to chemotherapy alone (70.1% vs. 53.3%; hr: 0.56; 95% ci: 0.36 to 0.85; *p* = 0.006), with an overall survival trend (hr: 0.65; 95% ci: 0.34 to 1.23; *p* = 0.18) 34. The hr and absolute benefit for the addition of trastuzumab are in the range seen during the large adjuvant trials with trastuzumab; however, these results were delivered with a much smaller number of patients (*n* = 227 vs. *n* > 13,000). The acceptable cardiac safety data also show that it may be reasonable to use a low cumulative dose of anthracycline concurrently with trastuzumab. Altogether, the noah trial has demonstrated that neoadjuvant trastuzumab for 1 year in combination with chemotherapy is a standard of care for women with labc or ibc her2-positive breast cancer. Publication of final results is eagerly awaited.

No trial has tested, or is testing, the effect of starting trastuzumab in the neoadjuvant setting against starting it after surgery. The ncctg 9831 trial has examined the benefits of trastuzumab concurrent with chemotherapy versus trastuzumab sequential after chemotherapy. Results of the data analysis are pending. In regard to duration, based on the adjuvant trials, total duration is 1 year at present. Studies to determine if a 6-month treatment is equivalent to a 12-month treatment (NCT00712140, NCT00615602) or if 2 years’ treatment provides further benefits over 1 year’s treatment (hera) are ongoing. In Finland, a trial is also comparing 9 weeks with 51 weeks of adjuvant trastuzumab (NCT00593697).

### 6.2 Possible Combination Regimens with Trastuzumab

#### Suggestion

Currently, the panel felt that concurrent trastuzumab with the taxane component of commonly-used neoadjuvant regimens is a reasonable standard in primary operable, locally advanced, or inflammatory breast cancer. However, current data are not strong enough to support the concurrent use of trastuzumab with anthracyclines, given the potential cardiac toxicity.

#### Discussion

The panel noted that no randomized trials were available in labc or ibc to support any particular trastuzumab-based regimen. Several published regimens included concurrent anthracyclines with trastuzumab 3,33,34,36, but caution is needed in using this approach because the overall sample size in these studies is small, the patient populations were highly selected, and the primary aim was not the cardiac safety of trastuzumab treatment given concurrently with anthracyclines. Also, the follow-up for cardiotoxicity was only short-term. It must be noted that the total dose of anthracyclines was lower than that usually given in the adjuvant setting, and the risk– benefit ratio of these regimens is not fully defined. Long-term follow-up and randomized comparisons of the regimens will be required for definitive recommendations. In the hera trial, higher cumulative doses of anthracyclines were found to contribute as a risk factor for trastuzumab-associated cardiotoxicity 42. Although the population benefiting from anthracyclines is that with *TOPO2A* gene alterations, found mainly in her2-positive breast cancers 43, no evidence supports concurrent anthracycline and trastuzumab in the adjuvant setting. In the metastatic setting, anthracyclines given concurrently have been shown to be associated with an unacceptable rate of cardiac toxicity, and therefore the combination is contraindicated in that setting 44.

In the context of primary operable breast cancer, in which patients seek a better chance for breast conservation, the panel agreed that all adjuvant data could be extrapolated to neoadjuvant therapy for clinical benefit, but with a higher chance of breast conservation. In the context of labc and ibc, there is increasing evidence that her2-targeted strategies used in the adjuvant setting may be applicable in the neoadjuvant setting. Data from the two randomized controlled trials of trastuzumab versus no trastuzumab in operable breast cancer (MD Anderson Trial) or in labc and ibc (noah trial) showed that trastuzumab is associated with a significant increase in pcr. In the absence of data from randomized trials comparing neoadjuvant with adjuvant trastuzumab, the panel felt that initiating trastuzumab preoperatively and continuing it postoperatively for 1 year in total is reasonable. In an ongoing phase iii trial in her2-positive breast cancer (Neo-Adjuvant Lapatinib and/or Trastuzumab Treatment Optimisation), the total duration of her2-targeted therapy is also 1 year.

Whether trastuzumab should be given concurrently with chemotherapy or after chemotherapy remains an open question. Answers will be obtained with the analysis of the ncctg 9831 trial in which trastuzumab was given concurrently with paclitaxel in one arm and trastuzumab was given sequentially in another arm in the adjuvant setting.

The transfer to the neoadjuvant setting of regimens proven efficacious in the adjuvant setting is supported by a meta-analysis 1. Although the regimens used were mainly doxorubicin and cyclophosphamide (ac), fac, and fe_60_c, it is reasonable to believe that other chemotherapy regimens would be as efficacious if given preoperatively compared with postoperatively. However, the optimal duration of chemotherapy is unknown. For example, the preliminary results of the nsabp 30 trial in operable breast cancer showed that 8 cycles of chemotherapy (ac followed by docetaxel) were superior to 4 cycles of doxorubicin and docetaxel or of docetaxel, doxorubicin, and cyclophosphamide 45.

Although the efficacy results are quite convincing, the panel warned that a decision to give concurrent neoadjuvant anthracyclines with trastuzumab could not be made based on the neoadjuvant trials conducted to date. More studies and longer follow-up from the aforementioned trials, with particular attention to cardiac effects, are required to provide further insight into the cardiac safety of such combinations.

## 7. CONCLUSIONS

Trastuzumab has become a standard of care in the adjuvant and metastatic settings for patients with her2-positive breast cancer. The evolving data in the neoadjuvant setting suggest that the addition of trastuzumab to neoadjuvant systemic therapy significantly increases pcr, breast conservation rates, and event-free survival. A strategy of concurrent anthracyclines with trastuzumab appears promising, possibly further maximizing the efficacy of trastuzumab in high-risk patient populations. However, the cardiotoxicity risk (although relatively low so far) continues to be a concern. The present article provided panel suggestions based on available data about the use of trastuzumab preoperatively. The suggestions set out here will continue to evolve as data and experience with trastuzumab and other her2-targeted agents in trials of neoadjuvant systemic therapy emerge.

## 8. DATE OF PANEL SUGGESTIONS

Panel suggestions were completed in June 2009.

## Figures and Tables

**TABLE I:** Key neoadjuvant trials of trastuzumab (h)

Reference	Treatment	Patients (*n*)	Pathologic cr [% (95% ci)]		dfs [% (95% ci)]	
			h arm	Non-h arm	h arm	Non-h arm
Buzdar *et al.,* 2005^3^ (MD Anderson trial)	h + (p→fec) vs. p→fec	42 (stage ii–iiia)	65.2 (43–84)	26.3 (9–51)	—	—
			p=0.016		—	
Buzdar *et al.,* 2007^33^,^a^ (MD Anderson trial update)		64	60.0 (44.3–74.3)	26.3 (9–51)	100 (85.2–100)^b^	85.3 (67.6–100)^b^
					100 (92–100)^c^	94.7 (85.2–100)^c^
			p value not reported		p= 0.041^b^	
Gianni *et al.,* 2008^34^,^d^,^e^ noah trial	h + (ap→p→cmf) →surgery→h vs. ap→p→cmf→surgery	227	43	23	70.1^f^	53.3^f^
			p=0.002		p=0.006 hr: 0.56	
Baselga *et al.,* 2007^35^ [noah trial (her2+ ibc)]		61	55	19	—	—
			p=0.004		—	

a Combined values, includes additional patients.

b 3-Year follow-up, does not include additional patients (*n*= 42).

c 1-Year follow-up, including additional patients.

d Overall survival data not yet mature. Preliminary results show 17 events in the h arm and 22 events in the non-h arm. There is a positive trend for the h arm at a median follow-up of 3 years: p = 0.18; hazard ratio: 0.65.

e 3-Year median follow-up.

f Presented as event-free survival, defined by either progression on therapy or relapse after surgery or death from any cause.

cr=complete response; ci= confidence interval; dfs= disease-free survival; p= paclitaxel; fec= 5-fluorouracil, epirubicin, cyclophosphamide; ap= doxorubicin, paclitaxel; cmf= cyclophosphamide, methotrexate, 5-fluorouracil; hr= hazard ratio; ibc= inflammatory breast cancer.

**TABLE II:** Future studies with her2–targeted therapy in the neoadjuvant setting

Trial name (ClinicalTrials.gov id)	Type	Treatment	Expected accrual	Estimated completion date
acosog-Z1041(NCT00513292)	Phase iii	fec_75_→th vs. th→fec_75_ + h	270	June 2008
EGF106988 (NCT00429299)	Phase ii	Chemotherapy + l vs. chemotherapy + h vs. chemotherapy + lh	120	June 2009
03–311 (NCT00148668)	Phase ii	h + vinorelbine vs. dcbh	81	December 2009
Neo altto (NCT00553358)	Phase iii	lh→lht vs. l→lt vs. h→ht	450	April 2010
calgb-40601 (NCT00770809)	Phase iii	h + t + l vs. h + t vs. t + l	400	June 2010
nsabp B-41 (NCT00486668)	Phase iii	ac→th vs. ac→tl vs. ac→thl	522	July 2010
GSK-LAP107087 (NCT00499681)	Phase ii	l + letrozole vs. placebo + letrozole	36	July 2010
1200.44 (NCT00826267)	Phase ii	bibw 2992 vs. l vs. h	120	December 2010
gbg 44 (NCT00567554)	Phase iii	ec→d vs. ec→d + b vs. t vs. t + everolimus vs. ec→dh vs. ec→dl	2547	December 2010
geicam/2006-14 (NCT00841828)	Phase ii	ec→d + l vs. ec→d + h	102	December 2011
EU-20851 (NCT00674414)	Phase ii	h vs. h + everolimus	120	April 2014
NeoSphere (NCT00545688)	Phase ii	h + d vs. h + p + d vs. h + p vs. p + d	400	August 2014
H-20464 (NCT00548184)	Phase ii	hl vs. hl + endocrine therapy	64	January 2015
LPT109096 (NCT00524303)	Phase ii	fec_75_→th vs. fec_75_→thl	99	Not available

f= 5-fluorouracil; e= epirubicin; c= cyclophosphamide; t= paclitaxel; h= trastuzumab; l= lapatinib; d= docetaxel; cb= carboplatin; a= doxorubicin; b= bevacizumab; p= pertuzumab.

## Footnotes

a Trademark of Genentech, San Francisco, CA, U.S.A.
